# Potential impact of platelet-to-lymphocyte ratio on prognosis in patients with colorectal cancer: A systematic review and meta-analysis

**DOI:** 10.3389/fsurg.2023.1139503

**Published:** 2023-03-27

**Authors:** Ganlin Guo, Xuhua Hu, Tianyi Gao, Huixian Zhou, Baokun Li, Chaoxi Zhou, Bin Yu, Guiying Wang

**Affiliations:** ^1^ The Second Hospital of Hebei Medical University, Shijiazhuang, China; ^2^ The Fourth Hospital of Hebei Medical University, Shijiazhuang, China; ^3^ The Third Hospital of Hebei Medical University, Shijiazhuang, China

**Keywords:** colorectal cancer, platelet-to-lymphocyte ratio, overall survival, progression-free survival, cancer-specific survival, disease-free survival, meta-analysis

## Abstract

**Background:**

Numerous studies have confirmed that inflammation promotes the occurrence, development and prognosis of colorectal cancer (CRC).

**Objective:**

This study focuses on the potentially prognostic value of the platelet-to-lymphocyte ratio (PLR) in CRC patients.

**Data Sources:**

This study was registered at PROSPERO (ID: CRD42020219215). Relative studies were searched on PubMed, Cochrane Library, Embase, Web of Science, and clinical trial databases by two back-to-back reviewers. *Study Selection and Intervention*: Studies were screened according to the predetermined inclusion and exclusion criteria, comparing prognosis differences between low PLR levels and high PLR levels for CRC patients. *Main Outcome Measures*: Studies were integrated and compared to analyze the value of PLR in predicting overall survival (OS), progression-free survival (PFS), cancer-specific survival (CSS), disease-free survival (DFS) and recurrence-free survival (RFS) of CRC. *Results*: Outcomes were compared using Review Manager (version 5.4) software from Cochrane Collaboration. A total of 27 literary works, including 13,330 patients, were incorporated into our study. The final results showed that higher PLR levels had worse OS (hazard ratio [HR] = 1.40, 95% confidence interval [CI] = 1.21–1.62, *P* < 0.00001), DFS (HR = 1.44, 95% CI = 1.09–1.90, *P* = 0.01) and RFS (HR = 1.48, 95% CI = 1.13–1.94, *P* = 0.005) than lower PLR levels, respectively. However, there was no evidence of significance for PFS (HR = 1.14, 95% CI = 0.84–1.54, *P* = 0.40) and CSS (HR = 1.16, 95% CI = 0.88–1.53, *P* = 0.28) in the final meta-analysis.

**Limitations:**

Our study has the following limitations. First of all, we only included literature published in English, which means that some publication bias may be inevitable. In addition, our study used aggregate data, not individual data; furthermore, we did not define the exact cut-off value representing the PLR level.

**Conclusion:**

An elevated PLR seems to be an adverse prognostic factor affecting survival outcomes in patients with CRC. Meanwhile, more prospective studies are required to confirm our conclusion.

**PROSPERO ID**: CRD42020219215.

## Introduction

Colorectal cancer (CRC) is the third most common cancer in the world and the second-leading cause of cancer-related death (1), the incidence of which shows a younger trend (2). At present, the diagnosis and treatment of CRC have been significantly improved. However, due to local tumor recurrence and metastasis, patients' long-term survival is poor, and OS and FPS are not ideal. It is predicted that the greatest impact and the fastest increase in the burden of cancer in the coming decades will continue to be in low- and middle-income countries, many of which are already facing overwhelming difficulties (3). Adding to the tension, current tumor markers, such as CEA and pik3, lack accuracy or are expensive. Therefore, it is particularly important to find an economical, convenient and effective biomarker to predict CRC prognosis.

Blood routine examination, including a series of indicators reflecting the level of inflammation, is simple and cheap but has not been paid attention to. As early as 1863, a German pathologist named Rudolf Virchow noticed “lymphoreticular infiltrate” in tumor tissue and linked inflammation to cancer (4). Additionally, immune cells are the main cellular components of tumor lesions and play an important role in the anti-tumor process (5), including CRC. The tumor microenvironment is the internal environment for tumor cell survival, which is composed of tumor cells, immune cells, cytokines, and cell metabolites. It has been reported that host cell interaction in the tumor microenvironment, such as immune cell infiltration, plays a crucial role in tumor progression and is closely related to patient prognosis (6). The platelet-to-lymphocyte ratio (PLR), as one of the evaluation indexes of systemic inflammatory response, not only can reflect the body's inflammatory response and is an independent risk factor for poor prognosis of various tumors, such as lung cancer, ovarian cancer, hepatocellular carcinoma, and so on (7–11) but also is available, inexpensive and repeatable in clinical laboratory examination. However, the relationship between the PLR and CRC remains controversial. Some studies reported that an elevated PLR was an independent adverse prognostic factor in patients with CRC (12–23), yet others showed that the PLR could not be a related prognostic biomarker of CRC (11, 24–35).

In this study, a meta-analysis was conducted on published survival data on the PLR and prognosis of CRC, and the relationship between different PLR levels and survival outcomes in patients with CRC was finally determined.

## Materials and methods

### Search strategy

This study was registered at PROSPERO (ID: CRD42020219215). Two authors independently searched relevant literature from PubMed, Cochrane Library, Embase, Web of Science, and clinical trial databases with the following search terms: (colorectal neoplasms or “CRC” or ((cancer* or carcinoma* or neoplasm* or adenoma* or adenocarcinom* or tumour* or tumor* or polyp* or malignan*) and (colorectal* or colon* or rect* or anal or anus or “large bowel”))) and (platelet to lymphocyte ratio or platelet lymphocyte ratio or “PLR”). We limited the research method to cohort studies, and the retrieval time was limited from the database construction to December 28, 2021. Subject words and random words were used during retrieval.

### Inclusion and exclusion criteria

Studies meeting the following criteria were included: (1) original articles published in English; (2) studies that compared prognosis in patients with CRC between different PLR levels; (3) patients were diagnosed with CRC by histopathological examination; (4) study methods were cohort studies; (5) studies that carried out multivariate analysis; (6) studies that succeeded in reporting the cut-off value of PLR and HR and a 95% CI. Studies were excluded when: (1) they were non-English studies; (2) they were non-cohort studies; (3) data could not be extracted from original articles; (4) studies failed to report the cut-off value of PLR or HR or a 95% CI; (5) original articles were letters, editorials, comments, supplements, conference abstracts, review articles, or duplicated and unrelated studies.

### Data extraction

Two researchers independently screened the literature, extracted the data, and cross-checked it. If there are differences, they will be discussed and resolved or handed over to a third researcher for judgment. For literature lacking information, we will try our best to contact the original author to supplement it. The extracted information is as follows: (1) basic information included in the study: first author’s name, year and country of publication, name of the journal, contact information; (2) basic characteristics of the study: sample size and age, no. of different PLR levels, follow-up time, TNM stage; (3) key elements of quality assessment; (4) main data of outcome indicators.

### Statistical analysis

We used Review Manager (version 5.4) software from Cochrane Collaboration for our meta-analysis, and *P* < 0.05 was considered statistically significant. HR and 95% CI were recorded to assess the strong connection between different PLR levels and CRC prognosis. The heterogeneity of included studies was analyzed and evaluated by combining the Cochrane Q test and *I*^2^ test (36). A *P*-value <0.1 for Q statistic test or *I*^2 ^> 50% was considered to be significantly heterogeneous. Then a random-effect model (DerSimonian–Laird method) was performed to calculate HR and 95% CI. Otherwise, a *P*-value >0.1 for Q statistic test or *I*^2^ < 50% was considered to use the fixed-effect model (Mantel–Haenszel method). However, it should be noted that when the number of studies is small, there is moderate or substantial heterogeneity and the distribution does not necessarily follow the normal or t-distribution. In this case, the random-effect models are preferred irrespective of the *I*^2^ statistic (37–40). When the number of included studies exceeded 10, funnel plots (41) were used to evaluate publication bias. Eventually, the stability and reliability of meta-analysis results were clarified through sensitivity analysis.

## Results

### Eligible articles

**Figure 1:**
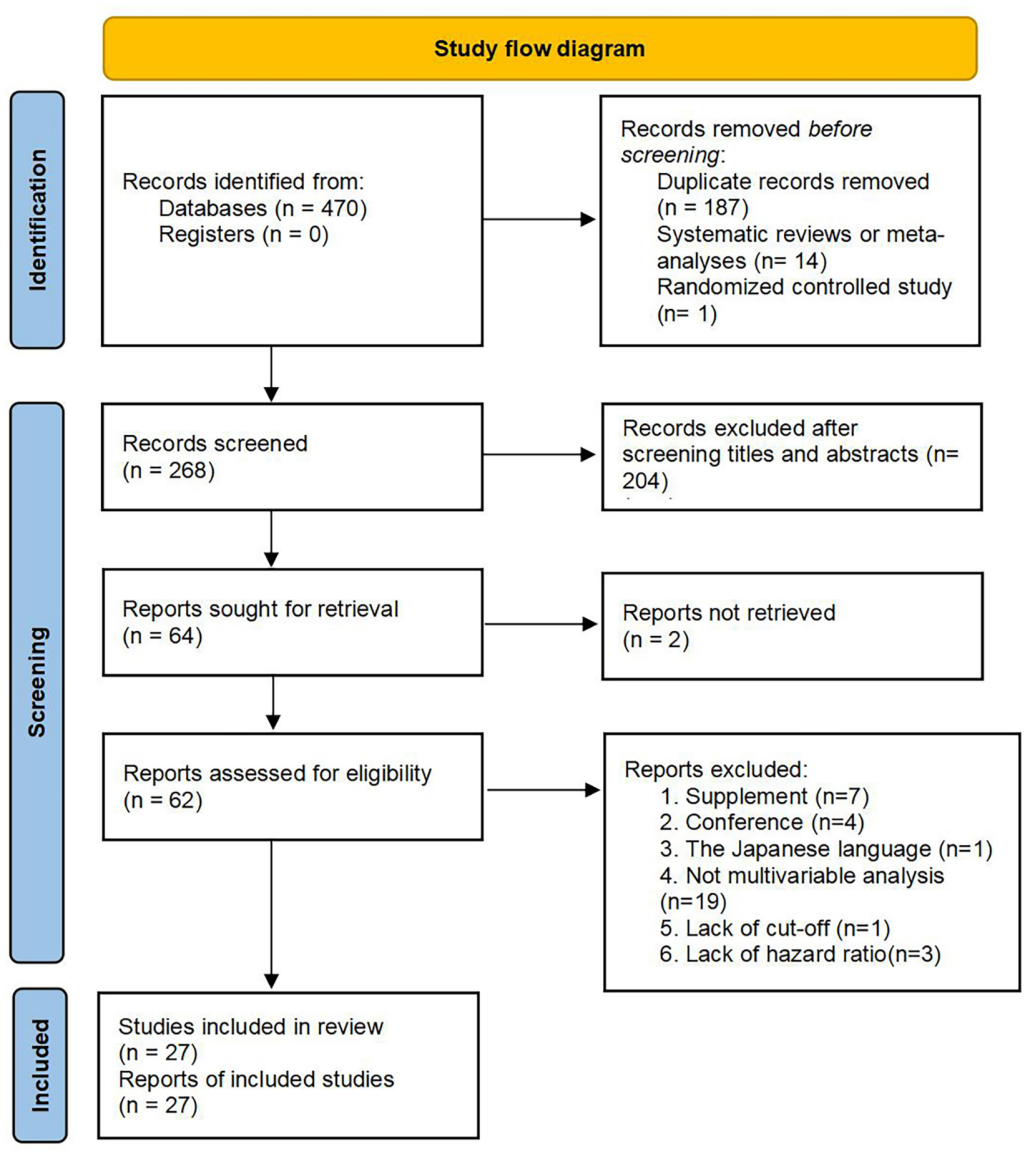
Flow diagram for study selection. *From:* Page MJ, McKenzie JE, Bossuyt PM, Boutron I, Hoffmann TC, Mulrow CD, et al. The PRISMA 2020 statement: an updated guideline for reporting systematic reviews. BMJ 2021;372:n71. doi: 10.1136/bmj.n71.

According to the aforementioned retrieval strategies, 470 literary works were identified, including 160 studies from PubMed, nine from Cochrane Library, 181 from Embase, 118 from Web of Science, and two from clinical trials. Because we obtained zero related literary works through other resources, 470 studies were finally screened. According to the inclusion and exclusion criteria, there were 187 duplicated records, 15 literary works with inconsistent research types (14 systematic reviews or meta-analyses, one randomized controlled study), two articles retrieved from clinical trials were unpublished, and 204 articles were removed after screening titles and abstracts. After that, after reviewing the full text of the remaining 62 articles, 35 records were excluded due to the following criteria: (1) supplement: *n* = 7; (2) conference: *n* = 4; (3) Japanese language: *n* = 1; (4) not multivariable analysis: *n* = 19; (5) a lack of cut-off: *n* = 1; (6) a lack of hazard ratio: *n* = 3. Consequently, there were 27 studies, including 13,330 CRC patients, included in our meta-analysis (Figure 1). In the included studies, there were 25 records for OS, 11 for PFS, five for CSS, three for DFS and three for RFS. Clinical characters and relevant information extraction of the eligible studies are shown in Table 1 (42–44).

**Table 1:** Main characteristics of included studies.

Study	Year	Country	Age (median and range)	Sample (male/female)	Survival analysis	HR (95% CI)	Treatment	Cut-off	No. of different level PLR	Stage	Summary results	NOS score
Jian-Hui Chen et al. (11)	2017	China	–	808/575	OS	R(M)	Resection	210	PLR ≤ 210 (low) and PLR > 210 (high)	I–IV	Positive(OS)	8
Ross D Dolan et al. (23)	2018	Britian	65/65–74/75:248/270/283	430/371	CSS;OS	R(M)	Resection	150	PLR ≤ 150 and PLR >150	I–IV	Negative(CSS); Negative(OS)	8
Zhigui Li et al. (45)	2019	China	62 (19–94)	176/136	OS;PFS	R(M)	Resection	260	PLR ≤ 260 and PLR >260	I–III	Positive(OS); Negative(PFS)	8
Mao-Song Lin et al. (12)	2017	China	61.38 ± 11.35 years (range, 33 to 82 years)	78/60	OS	R(M)	colonoectomy	248	PLR ≤ 248 and PLR >248	I–IV	Positive(OS)	9
Chong Lu et al. (13)	2017	China	<60/≥60:817/1028	1044/801	OS;CSS	R(M)	surgery	130	PLR ≤ 130 and PLR >130	I–IV	Positive(OS); Positive(CSS)	9
Yongxi Song et al. (24)	2017	China	62 (range 13–86)	982/762	OS;CSS	M	Resection	134.6	PLR < 134.6 and PLR ≥134.6	I–IV	Negative(OS); Negative(CSS)	9
Lin Wang et al. (14)	2020	China	20 to 67	235/164	OS	M	Resection	–	–	III	Positive(OS)	7
Hou-Qun Ying et al. (10)	2014	China	<60/≥60:70/135	144/61	RFS/OS/CSS	M	Resection	176	PLR < 176 and PLR ≥176	I–III	Negative(RFS);Negative(OS);Negative(CSS)	8
Joanna Szkandera et al. (25)	2014	Austria	64 (27–95)	217/155	OS	M	Resection	225	PLR ≤ 225) / PLR225/Unknown:192/164/16	II–III	Negative(OS)	9
Yuka Ahiko et al. (26)	2021	Japan	65 years (25%–75% interquartile range, 57–72 y)	1111/769	OS	M	Resection	136	PLR < 136)/PL ≥ 136:759/1121	II–III	Negative(OS)	9
Ozawa et al. (15)	2015	Japan	<65/≥65:78/156	142/92	DFS/CSS	M	Resection	25.4	PLR <25.4 (181) and PLR ≥ 25.4 (53)	II	Positive(DFS); Positive(CSS)	8
Özlem Mermut et al. (27)	2020	Turkey	61 (range: 22–83)	85/71	OS	M	Resection	192	PLR < 192 (84) and PLR ≥ 192 (72)	II–III	Negative(OS)	7
Dongyan Cai et al. (16)	2019	China	62.0 ± 10.6 years	86/78	OS	M	–	136		I–IV	Positive(OS)	7
Ender Dogan et al. (28)	2019	Turkey	61 (26–82)	66/64	PFS/OS	M	Not clear	160.66	PLR <160.66 (low) and PLR ≥ 160. 66 (high)	IV	Negative(PFS)/Negative(OS)	6
Akihisa Matsuda et al. (29)	2019	Japan	69 (48–90)	20/13	PFS/OS	M	Not clear	173.2	–	IV	Negative(PFS)/Negative(OS)	9
Yuchen Wu et al. (46)	2016	China	59	35/20	OS/PFS	M	Not clear	150	PLR < 150 (31) / PLR ≥ 150 (24)	IV	Negative(OS)/Positive(PFS)	8
Jing Yang et al. (30)	2017	China	56 years (range 27–86)	58/37	PFS/OS	M	treated with cetuximab	142	PLR < 142 / PLR ≥ 142	IV	Negative(PFS)/Negative(OS)	8
Akihisa Matsuda et al. (17)	2020	Japan	–	15/6	PFS	M	Treated With Aflibercept plus FOLFIRI	193.2	cutoff: 193.2. high/low:11/10	IV	Positive(PFS)	9
Alessandro Passardi et al. (31)	2016	Italy	Low 66 (33–83) High 65 (34–81)	174/115	PFS/OS	M	–	169	PLR, low (<169) / high (≥169):144:145	I–IV	Negative(PFS)/Negative(OS)	7
Heming Li et al. (32)	2021	China	63 (36–89)	30/16	PFS	M	first-line therapy	117.81	PLR, low (<117.81) / high (≥117.81)	IV	Negative(PFS)	7
Jae Hyun Kim et al. (18)	2017	Korea	65	1027/796	OS/DFS	M	–	160	PLR, low (<160) / high (≥160):973/894	III/IV	Positive(OS)/Positive(DFS)	9
Derek J. Erstad et al. (19)	2020	USA	58	84/67	OS	M	hepatic resection	220	PLR, low (<220) / high (≥220)	IV	Positive(OS)	7
Marlid Cruz-Ramos et al. (20)	2019	Spain	72.2	63/47	OS/PFS	M	–	172.4	PLR, low (<172.4) / high (≥172.4)	IV	Positive(OS)/Positive(PFS)	7
Gulcan Bulut et al. (33)	2022	Turkey	64 years (32–84 years)	56/38	OS/PFS	M	–	180.36	PLR, ≥ 180.36/<180.36:47/47	IV	Negative(OS)/Negative(PFS)	6
Yan-Yan Wang et al. (34)	2019	China	57 years (IQR 50–64)	289/163	OS/DFS	M	–	186	PLR, low (<186) / high (≥186)	IV	Negative(OS)/Negative(DFS)	8
Joey Mercier et al. (21)	2019	Canada	–	95/57	PFS/OS	M	–	330	PLR <330 (112) and PLR ≥ 330 (40)	IV	Positive(PFS)/Positive(OS)	7
Martin Bailon-Cuadrado et al. (22)	2018	Spain	69.09 ± 11.18 years	114/87	OS/RFS	M	Resection	153	High PLR (*n* = 96)/Low PLR (*n* = 105)	IV	Positive(OS)/Positive(RFS)	7

### Quality assessment

After extracting data from the original articles, the Newcastle-Ottawa Scale (NOS) was used to assess the methodological quality of included studies (22, 45). The NOS is a simple and convenient quality assessment tool widely used to evaluate case control and cohort studies. The NOS consists of three parts: cohort selection, comparability, and results. Each part has evaluation items, each item is represented by ⋆, and the full score is 9. Our quality evaluation table is revealed in Table 2.

**Table 2:** Newcastle-Ottawa Scale for assessing the quality of the included studies

Study	Year	Representativeness of the exposed cohort	Selection of the nonexposed cohort	Ascertainment of exposure	Demonstration that outcome of interest was not present at start of study	Comparability of cohorts on the basis of the design or analysis	Assessment of outcome	Was follow-up long enough for outcomes to occur	Adequacy of follow up of cohorts	Totall scores
Jian-Hui Chen et al.	2017	⋆	⋆	⋆	⋆	⋆⋆	⋆	⋆		8
Ross D Dolan et al.	2018	⋆	⋆	⋆	⋆	⋆⋆	⋆	⋆		8
Zhigui Li et al.	2019	⋆	⋆	⋆	⋆	⋆⋆	⋆	⋆		8
Mao-Song Lin et al.	2017	⋆	⋆	⋆	⋆	⋆⋆	⋆	⋆	⋆	9
Chong Lu et al.	2017	⋆	⋆	⋆	⋆	⋆⋆	⋆	⋆	⋆	9
Yongxi Song et al.	2017	⋆	⋆	⋆	⋆	⋆⋆	⋆	⋆	⋆	9
Lin Wang et al.	2020	⋆	⋆	⋆	⋆	⋆⋆		⋆		7
Hou-Qun Ying et al.	2014	⋆	⋆	⋆	⋆	⋆⋆	⋆	⋆		8
Joanna Szkandera et al.	2014	⋆	⋆	⋆	⋆	⋆⋆	⋆	⋆	⋆	9
Yuka Ahiko et al.	2021	⋆	⋆	⋆	⋆	⋆⋆	⋆	⋆	⋆	9
Tsuyoshi Ozawa et al.	2015	⋆	⋆	⋆	⋆	⋆⋆	⋆	⋆		8
Özlem Mermut et al.	2020	⋆	⋆	⋆	⋆	⋆⋆		⋆		7
Dongyan Cai et al.	2019	⋆	⋆	⋆	⋆	⋆⋆		⋆		7
Ender Dogan et al.	2019	⋆	⋆	⋆	⋆	⋆⋆				6
Akihisa Matsuda et al.	2019	⋆	⋆	⋆	⋆	⋆⋆	⋆	⋆	⋆	9
Yuchen Wu et al.	2016	⋆	⋆	⋆	⋆	⋆⋆	⋆	⋆		8
Jing Yang et al.	2017	⋆	⋆	⋆	⋆	⋆⋆		⋆	⋆	8
Akihisa Matsuda et al.	2020	⋆	⋆	⋆	⋆	⋆⋆	⋆	⋆	⋆	9
Alessandro Passardi et al.	2016	⋆	⋆	⋆	⋆	⋆⋆		⋆		7
Heming Li et al.	2021	⋆	⋆	⋆	⋆	⋆⋆		⋆		7
Jae Hyun Kim et al.	2017	⋆	⋆	⋆	⋆	⋆⋆	⋆	⋆	⋆	9
Derek J. Erstad et al.	2020	⋆	⋆	⋆	⋆	⋆⋆		⋆		7
Marlid Cruz-Ramos et al.	2019	⋆	⋆	⋆	⋆	⋆⋆	⋆			7
Gulcan Bulut et al.	2021	⋆	⋆	⋆	⋆	⋆⋆				6
Yan-Yan Wang et al.	2019	⋆	⋆	⋆	⋆	⋆⋆	⋆	⋆		8
Joey Mercier et al.	2019	⋆	⋆	⋆	⋆	⋆⋆	⋆			7
Martin Bailon-Cuadrado et al.	2018	⋆	⋆	⋆	⋆	⋆⋆		⋆		7

### OS, DFS, RFS and PLR

**Figure 2:**
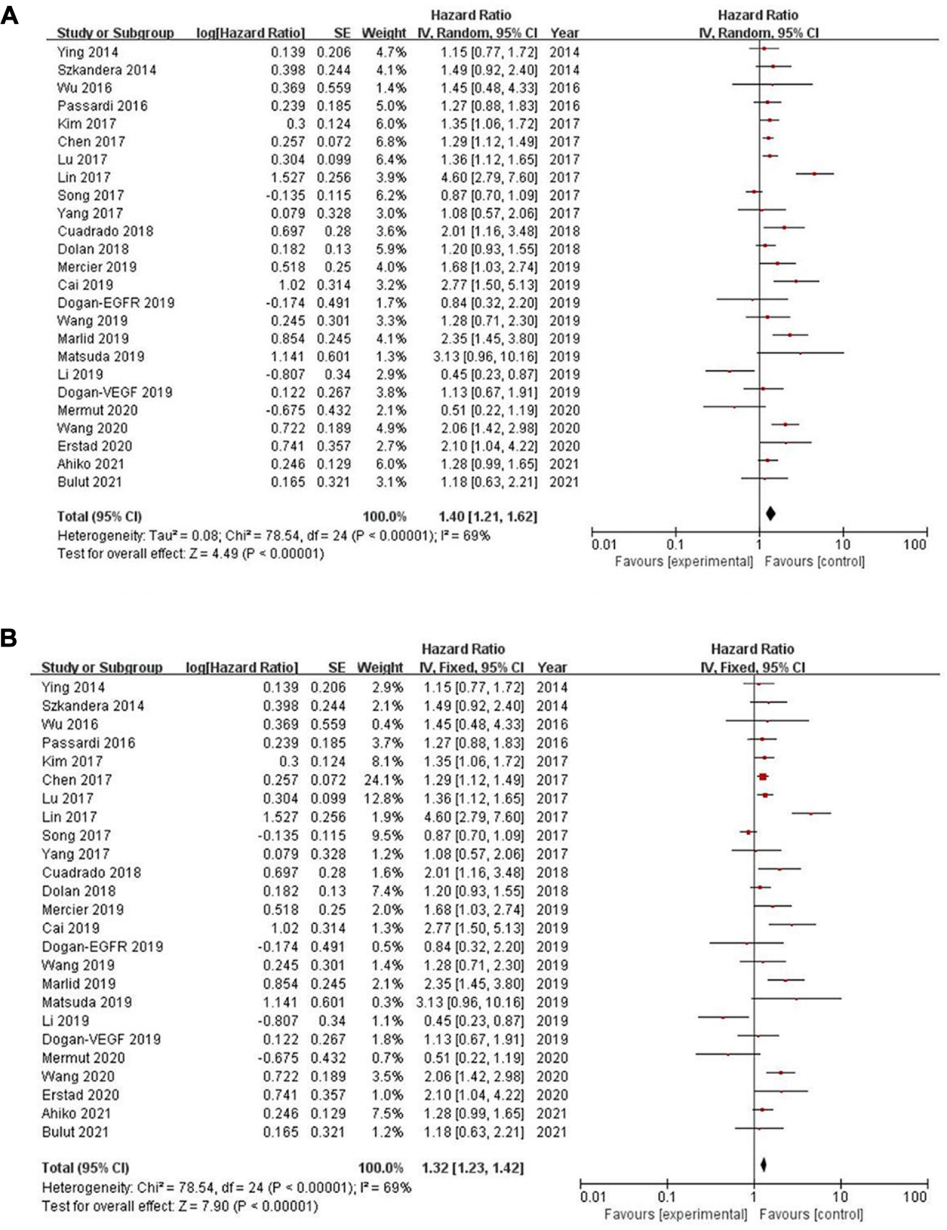
Forest graph on association of overall survival with different levels of platelet-to-lymphocyte ratio groups. (**A**) random effect model. (**B**) fixed effect model.

**Figure 3:**
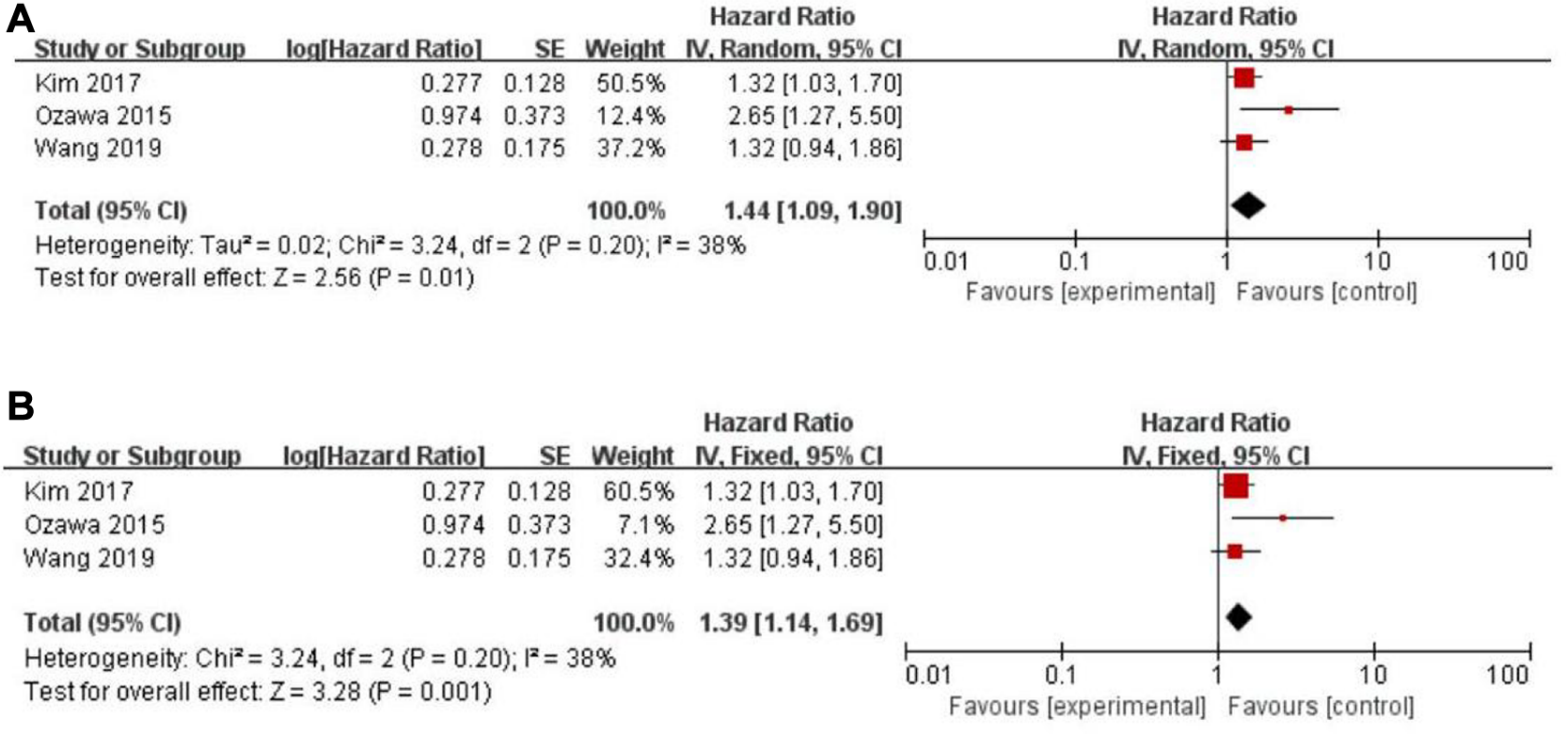
Forest graph on association of disease-free survival with different levels of platelet-to-lymphocyte ratio groups. (**A**) random effect model. (**B**) fixed effect model.

**Figure 4:**
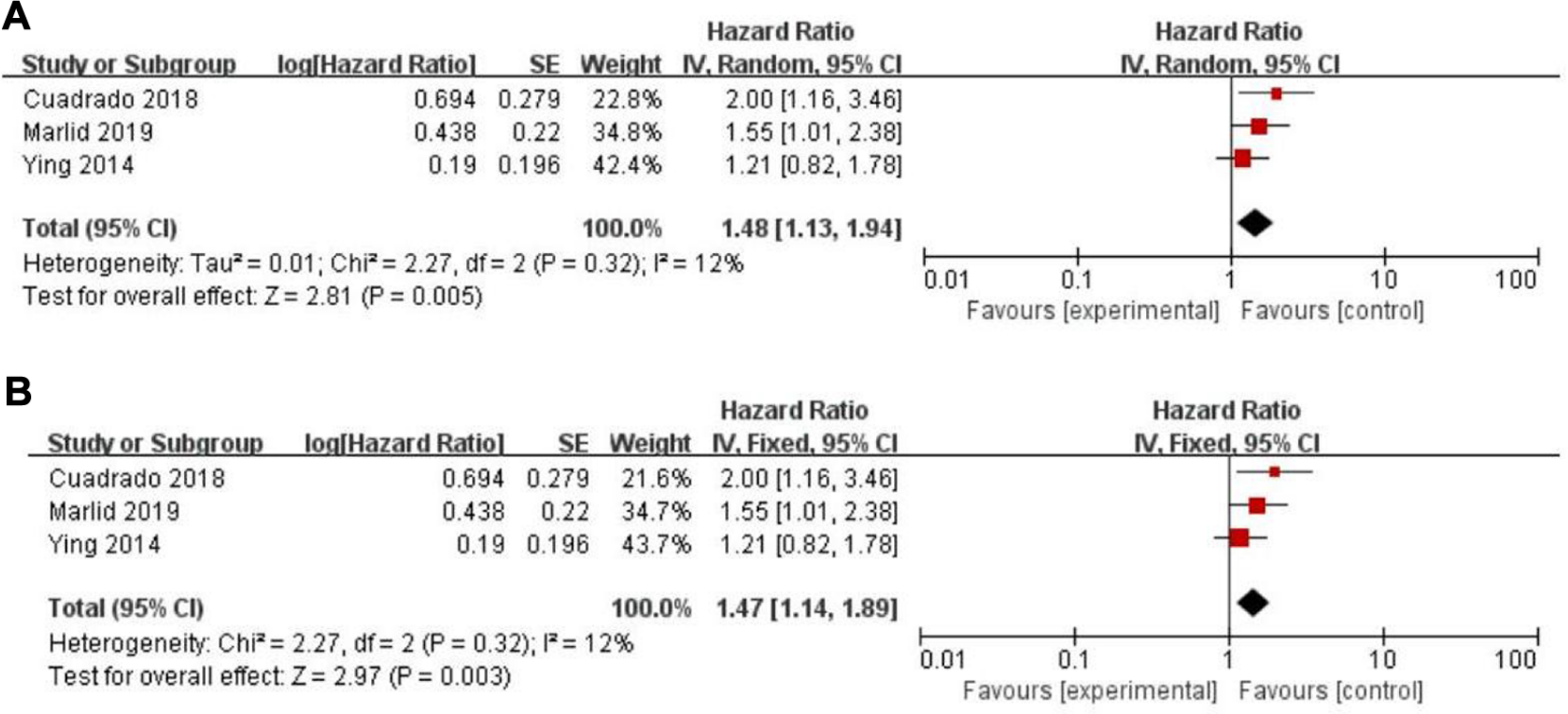
Forest graph on association of recurrence-free survival with different levels of platelet-to-lymphocyte ratio groups. (**A**) random effect model. (**B**) fixed effect model.

Twenty-five of the included records reported that overall survival (OS) of CRC patients with high PLR levels was significantly shorter than for those with low PLR levels (11–15, 17, 19–27, 29–31, 34, 35, 46–50). Pooled analysis of OS revealed significant differences and moderate heterogeneity between the high and low PLR groups in the random-effect model (HR = 1.40, 95% CI = 1.21–1.62, *P* < 0.00001, Figure 2A). Meanwhile, there were three studies evaluating hazard ratios for disease-free survival (DFS) (16, 19, 35) and recurrence-free survival (RFS) (11, 21, 23), respectively. As for DFS and RFS only few studies were available, considering that between-trial heterogenicity was still significant, random effect-models were preferred, and an elevated PLR was related to poor prognosis of patients with CRC for DFS and RFS (HR = 1.44, 95% CI = 1.09–1.90, *P* = 0.01 and HR = 1.48, 95% CI = 1.13–1.94, *P* = 0.005, respectively, Figures 3A, 4A).

### PFS, CSS and PLR

**Figure 5:**
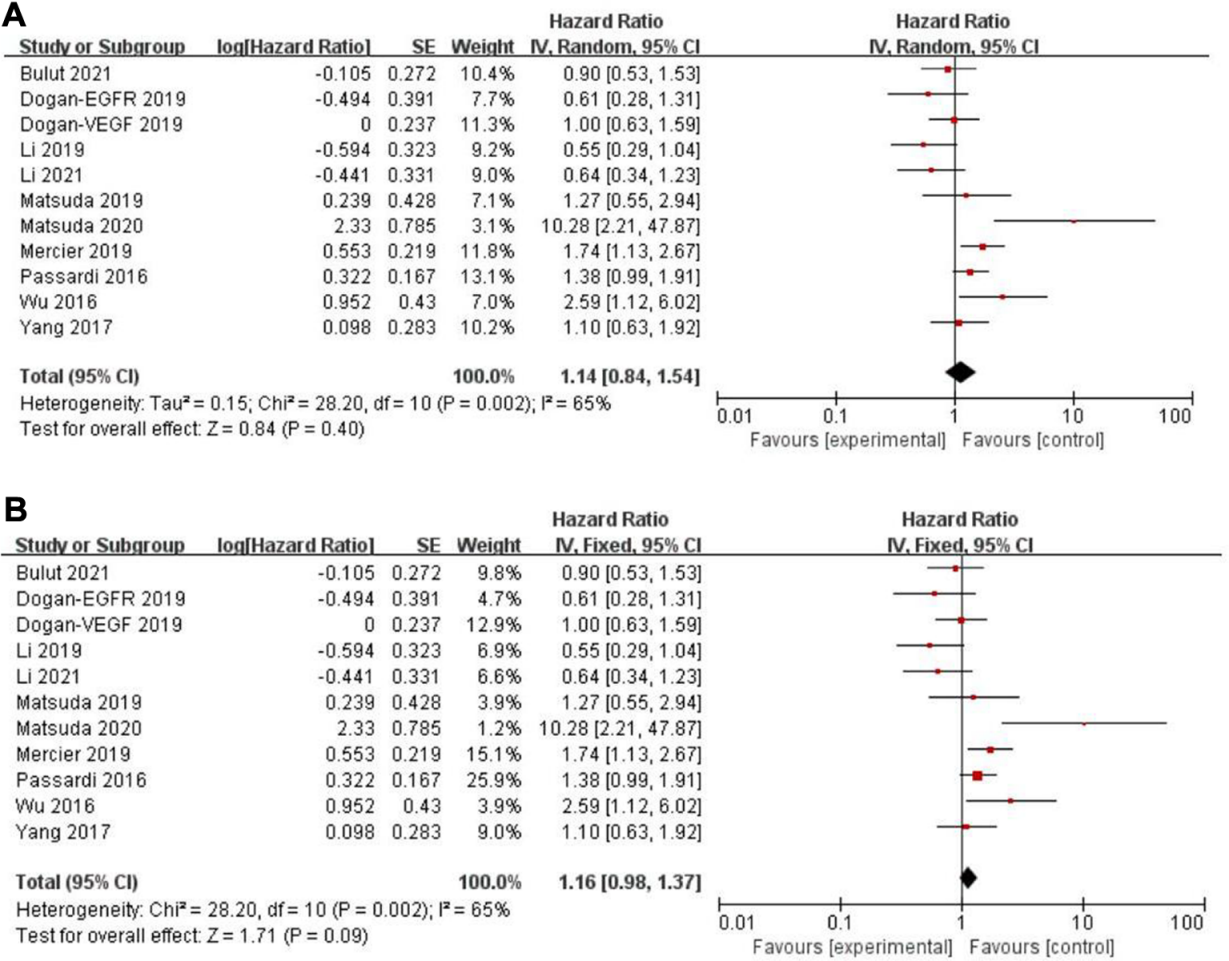
Forest graph on association of progression-free survival with different levels of platelet-to-lymphocyte ratio groups. (**A**) random effect model. (**B**) fixed effect model.

**Figure 6:**
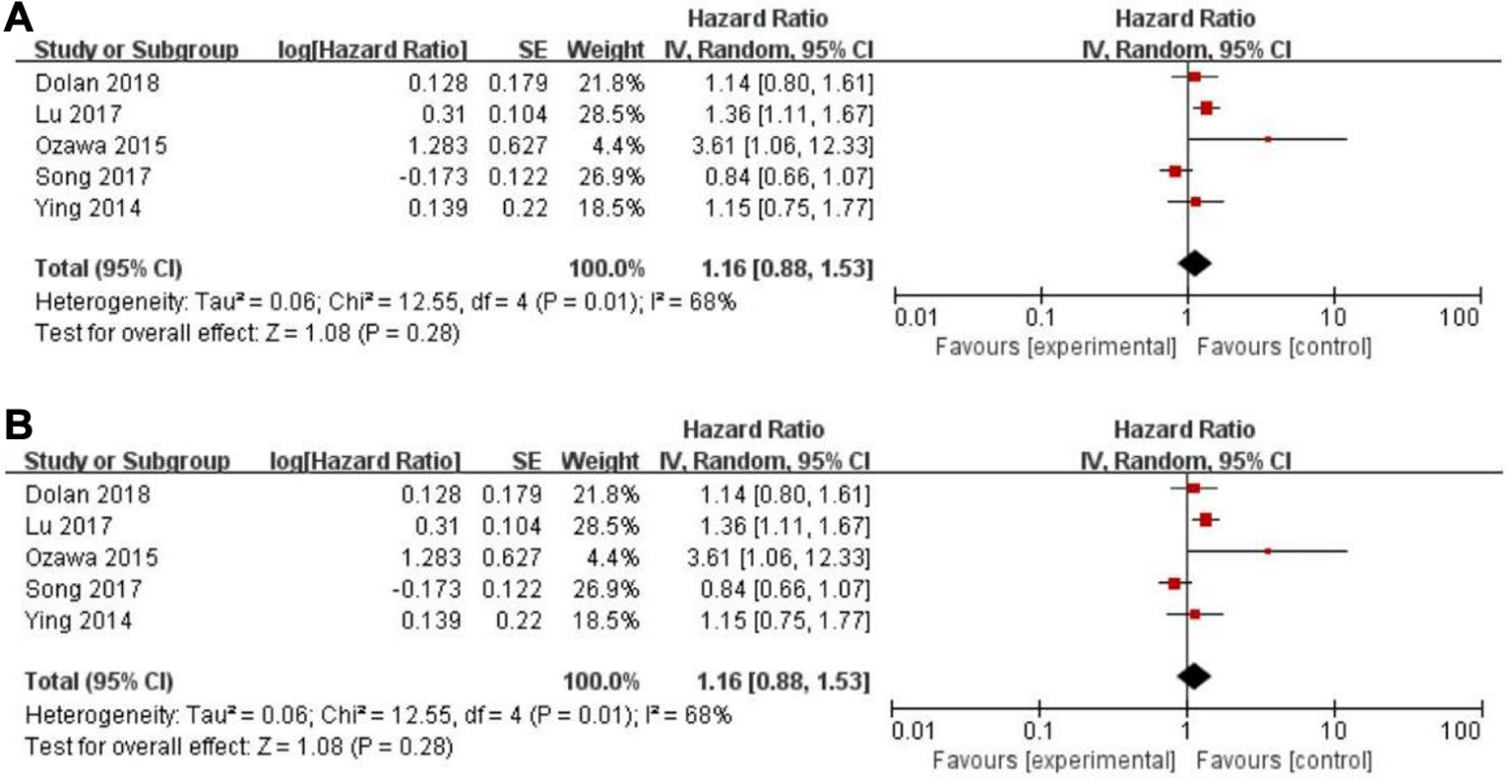
Forest graph on association of cancer-specific survival with different levels of platelet-to-lymphocyte ratio groups. (**A**) random effect model. (**B**) fixed effect model.

Eleven records assessed progression-free survival (PFS) (18, 29–31, 33, 34, 46, 48, 49, 50), whereas a non-significant association between PFS and the PLR (HR = 1.14, 95% CI = 0.84–1.54, *P* = 0.40; *I*^2^ = 65%, PH = 0.002, Figure 5A) was observed. Another five articles evaluated cancer-specific survival (CSS) (11, 14, 16, 25, 49). However, there was also no evidence of significance for CSS and PLR (HR = 1.16, 95% CI = 0.88–1.53, *P* = 0.28; *I*^2 ^= 68%, PH = 0.01, Figure 6A).

### Sensitivity analysis

The purpose of sensitivity analysis is to determine the stability and reliability of meta-analysis results. in OS, PFS and CSS tests, random-effect models were adopted according to Q test, so the fixed-effect models were used for sensitivity analysis (51). The results of OS, PFS and CSS (HR = 1.32, 95% CI = 1.23–1.42, *P* < 0.00001, HR = 1.16, 95% CI = 0.98–1.37, *P* = 0.09 and HR = 1.16, 95% CI = 0.88–1.53, *P* = 0.28, respectively, Figures 2B, 5B, 6B) were stable and reliable. In DFS and RFS, the results (HR = 1.39, 95% CI = 1.14–1.69, *P* = 0.001 and HR = 1.47, 95% CI = 1.14–1.89, *P* = 0.003, respectively, Figures 3B, 4B) were consistent with those of the original random-effect model, indicating that the meta-analysis results of DFS and RFS were also stable and reliable.

### Publication bias assessment

#### OS and PFS

**Figure 7:**
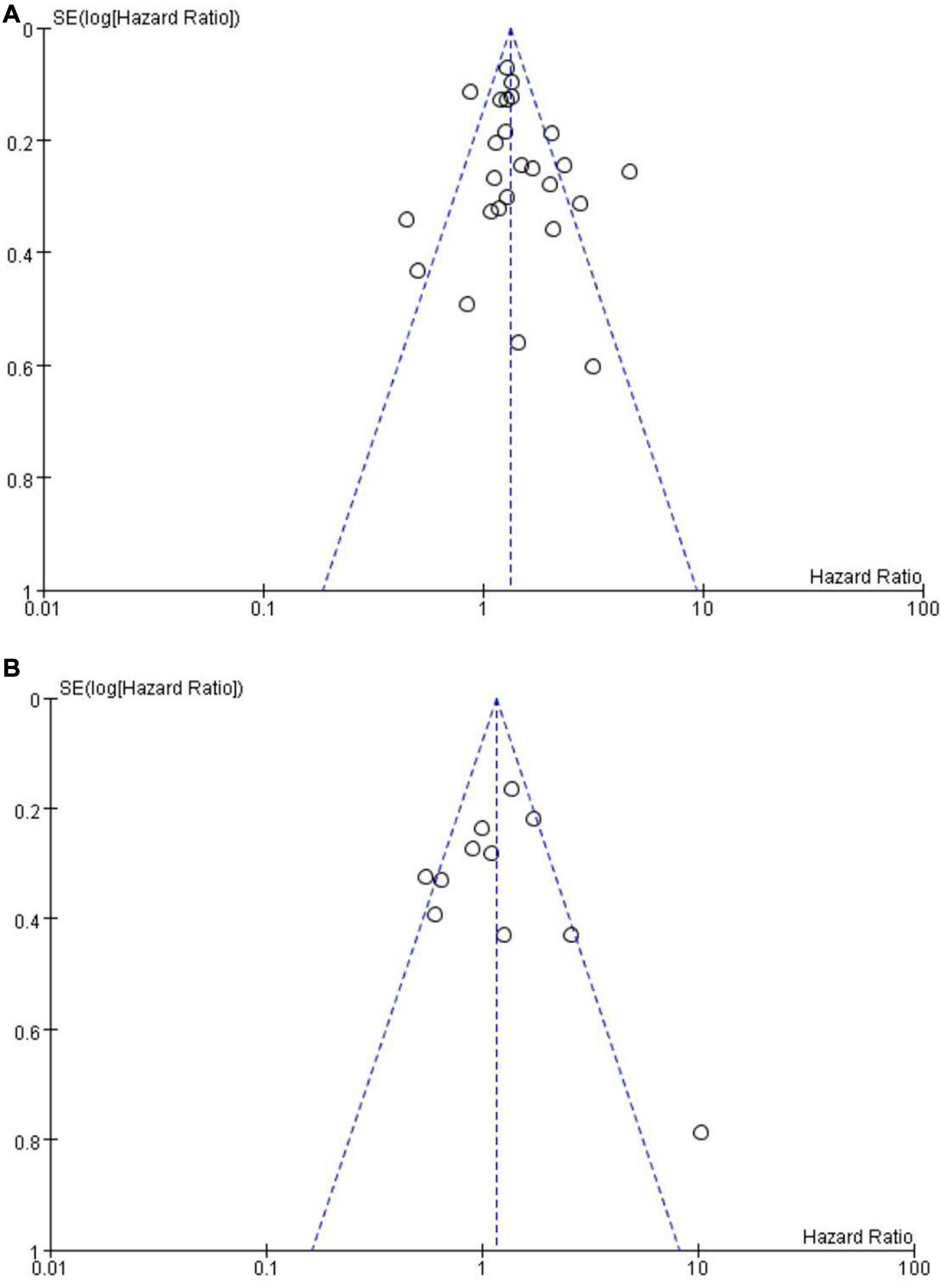
Funnel plot on the association of overall survival (**A**) and progression-free survival (**B**) with different levels of platelet-to-lymphocyte ratio groups.

Since more than ten studies were included in the OS group and the PFS group, a funnel plot was used to evaluate publication bias (Figures 7A,B). The funnel plots for publication bias show no obvious asymmetry, indicating that the pooled results were not influenced by publication bias.

#### DFS, RFS and CSS

Because of the small number of included studies, funnel plots have not been carried out.

## Discussion

Colorectal cancer is the third most common malignant tumor in the world and the second largest cause of cancer-related deaths. In 2018, there were about 1.8 million new cases and 881,000 deaths worldwide (52). The early clinical manifestations of CRC are not typical, and some patients have local progression or metastasis when found, leading to poor survival prognosis. Deeply studying the factors related to the prognosis of CRC and actively looking for tumor markers related to the prognosis of CRC are helpful to guide clinicians to evaluate the prognosis of CRC patients (53). However, two commonly used blood biomarkers, carcinoembryonic antigen (CEA) and carbohydrate antigen 19-9 (CA19-9), are not highly sensitive and specific (54). Some new tumor biomarkers such as circulating tumor cells (CTC) and circulating tumor DNA (ctDNA) (55) are also faced with high detection costs. Therefore, finding and screening some new, simpler and effective molecular indicators related to the prognosis of CRC is still a hot topic that needs further study.

Nowadays, an increasing number of researchers have confirmed that the platelet-to-lymphocyte ratio (PLR), the neutrophils-to-lymphocyte ratio (NLR) and the lymphocyte-to-monocyte ratio (LMR), as the common inflammatory indicators, are of great significance in the diagnosis and prognosis of tumors, including CRC (56, 57). The relationship between PLR and the prognosis of CRC has attracted our attention. Then, we explored the prognostic effect of PLR in 27 studies, including 13,330 patients with CRC. In our study, we searched the literature as comprehensively as possible, consisting of PubMed, Cochrane Library, Embase, Web of Science, and clinical trial databases. Our primary outcomes were OS and PFS, and secondary outcomes included CSS, DFS and RFR. We used Review Manager (version 5.4) software from Cochrane Collaboration for our meta-analysis, and *P* < 0.05 was considered statistically significant. The heterogeneity of included studies was mainly analyzed and evaluated by combining the Cochrane Q test and *I*^2^ test. Thus, in OS, PFS and CSS tests, random-effect models were adopted. As for DFS and RFS only three studies were available respectively, considering that between-trial heterogenicity was still significant, we similarly used random effect-models in the analysis of them. What's more, taking the uncertainty in the estimation of the between-trial heterogeneity, the fixed-effect models were used to verify the results. Eventually, our pooled results demonstrated that an elevated PLR was correlated with poor OS, DFS and RFS, which were consistent with those of previous meta-analysis (58–60). Furthermore, among them, we included more relevant articles, obtained more data and used more stringent methods.

Investigating the reason, PLR is one of the indicators reflecting the body’s inflammatory response, and studies have indicated that inflammation is related to the occurrence and development of tumors to some extent (61). Inflammatory cells can release a variety of bioactive substances in the tumor microenvironment, and the tumor microenvironment is the internal environment for tumor cell survival, which is composed of tumor cells, immune cells, cytokines, and cell metabolites. Simultaneously, various cells interact with inflammatory factors, which further promote the formation of tumors due to the extreme immunosuppressive microenvironment (62). Meanwhile, several studies have pointed out that platelets can secrete *P*-selectin adhesion factor, which can make inflammatory cells adhere to endothelial cells and have a great adverse impact on tumor production, metastasis and prognosis (63–65). In addition, the angiogenic factors released by platelets can promote endothelial cell growth, induce the proliferation and migration of endothelial cells, increase vascular permeability of tissue, and facilitate the invasion and metastasis of tumor cells through blood vessels in the body (66). Moreover, platelets can release tumor growth-promoting factors, resulting in continuous crazy growth of tumor tissue, which affects patient prognosis (67–69). Therefore, in CRC progression, PLR levels will gradually increase, and the higher the PLR, the worse the prognosis.

At the same time, it is interesting to note that under the assistant of R language, several new statistical research methods such as Paule-Mandel (70), Hartung-Knapp-Sidik-Jonkman (19) etc are developing with each passing day. Ralf Bender et al. (71) simultaneously pointed out that DerSimonian and Laird approach had some limitations, especially in the case of very few studies, and they recommend using Knapp-Hartung method for meta-analyses. It can be seen that everyone is working hard to eliminate the between-trial heterogeneity. And we known this and made our own efforts. Henmi et al. (72) use of fixed effects estimates with a random effects approach to deal with heterogeneity. Böhning D et al. (73) mentioned that if there is strong heterogeneity, the results of the random effect model and the fixed effect model will differ greatly; If there is small heterogeneity, the both approaches will provide similar results. Based on this, in our study, when the random effect models were used for data analysis, the fixed effect models were used to verify the results. The same results are obtained in the statistical results, which mean that the conclusion is stable and reliable. Considering that the heterogeneity between independent studies has not had a huge impact on the production of results and conclusions, nor has it produced new controversial results and conclusions, we have not introduced more complex statistical methods or analysis programs. Of course, we agree that these new statistical methods are very interesting and useful, and we will try to use them in the future.

Our results can better reflect the authenticity of the relationship between PLR and CRC prognosis. However, our study has the following limitations. First of all, we only included literature published in English, which means that some publication bias may be inevitable. In addition, our study used aggregate data, not individual data; furthermore, we did not define the exact cut-off value representing the PLR level. It is really a pity that there is no way to obtain the original data for statistics and determine the exact cut-off value. Because many articles give different cut-off values, we can only try to use “high level” and “low level” to evaluate. At the same time, as an analogy, similar problems have been encountered in similar studies conducted by predecessors. Hamid et al. (74) showed that a low lymphocyte-to-monocyte ratio, but not a high platelet-to-lymphocyte ratio, was inversely correlated with complete pathologic response rate. In his another research about prognostic value of neurophil-to-lysophocyte ratio (NLR) after curative rectal cancer recovery, result indicated that high NLR was associated with worse OS (75). It can be seen that the determination of the clear cut-off value of similar research did puzzling everyone. In addition, in order to avoid the impact of such differences as much as possible, we used both “random effect model” and “fixed effect model” in statistics, and the results are consistent. we know that our research is not perfect, but it provides a relatively useful result for clinical application. we also look forward to high-quality, multi-center RCT research to eliminate these deviations and determine the exact cut-off value.

Despite these limitations, our meta-analysis assessed the impact of an elevated PLR upon OS, PFS, CSS, DFS, and RFS outcomes in CRC patients. Based on existing data, our study suggests that PLR has a certain correlation with CRC patient prognosis, and an elevated PLR is correlated with poorer OS, DFS and RFS. However, no evidence exists that an elevated PLR is significantly associated with worse PFS and CSS. Clinical workers strengthening the detection of this available, inexpensive and repeatable index will have a good reference value for CRC patients. Moreover, with the emergence of more and more convincing studies, the impact of an elevated PLR upon CRC patient prognosis can be further explored and confirmed.

## Conclusions

An elevated PLR seems to be an adverse prognostic factor affecting survival outcomes in CRC patients. Meanwhile, more prospective studies are required to confirm our conclusion.

## Data Availability

The original contributions presented in the study are included in the article/Supplementary Material, further inquiries can be directed to the corresponding author.
